# Comparative Toxicity of Aquatic Per‐ and Polyfluoroalkyl Substance Exposure in Three Species of Amphibians

**DOI:** 10.1002/etc.5319

**Published:** 2022-03-31

**Authors:** R. Wesley Flynn, Gary Hoover, Michael Iacchetta, Samuel Guffey, Chloe de Perre, Belinda Huerta, Weiming Li, Jason T. Hoverman, Linda Lee, Maria S. Sepúlveda

**Affiliations:** ^1^ Upper Midwest Environmental Sciences Center US Geological Survey La Crosse Wisconsin USA; ^2^ Department of Forestry & Natural Resources Purdue University West Lafayette Indiana USA; ^3^ Department of Agronomy Purdue University West Lafayette Indiana USA; ^4^ Department of Fisheries & Wildlife Michigan State University Lansing Michigan USA; ^5^ School of Chemical Sciences Dublin City University Dublin Ireland; ^6^ Faculty of Life Sciences Universidad Andres Bello Santiago Chile

**Keywords:** Amphibians, Aquatic toxicology, Perfluoroalkyl substances

## Abstract

Per‐ and polyfluoroalkyl substances (PFAS) are contaminants of concern due to their widespread occurrence in the environment, persistence, and potential to elicit a range of negative health effects. Per‐ and polyfluoroalkyl substances are regularly detected in surface waters, but their effects on many aquatic organisms are still poorly understood. Species with thyroid‐dependent development, like amphibians, can be especially susceptible to PFAS effects on thyroid hormone regulation. We examined sublethal effects of aquatic exposure to four commonly detected PFAS on larval northern leopard frogs (*Rana [Lithobates] pipiens*), American toads (*Anaxyrus americanus*), and eastern tiger salamanders (*Ambystoma tigrinum*). Animals were exposed for 30 days (frogs and salamanders) or until metamorphosis (toads) to 10, 100, or 1000 μg/L of perfluorooctane sulfonate (PFOS), perfluorooctanoic acid (PFOA), perfluorohexane sulfonate (PFHxS), or 6:2 fluorotelomer sulfonate (6:2 FTS). We determined that chronic exposure to common PFAS can negatively affect amphibian body condition and development at concentrations as low as 10 µg/L. These effects were highly species dependent, with species having prolonged larval development (frogs and salamanders) being more sensitive to PFAS than more rapidly developing species (toads). Our results demonstrate that some species could experience sublethal effects at sites with surface waters highly affected by PFAS. Our results also indicate that evaluating PFAS toxicity using a single species may not be sufficient for accurate amphibian risk assessment. Future studies are needed to determine whether these differences in susceptibility can be predicted from species' life histories and whether more commonly occurring environmental levels of PFAS could affect amphibians. *Environ Toxicol Chem* 2022;41:1407–1415. © 2022 The Authors. *Environmental Toxicology and Chemistry* published by Wiley Periodicals LLC on behalf of SETAC.

## INTRODUCTION

Per‐ and polyfluoroalkyl substances (PFAS) are a large family (~5000) of widely used legacy and emerging contaminants that are pervasive in aquatic habitats globally and thus present a risk to the health of humans and wildlife (Agency for Toxic Substances and Disease Registry, 2018). Per‐ and polyfluoroalkyl substances are integral to many consumer and industrial products including aqueous film‐forming foams (AFFFs; Ahrens et al., 2015; Zhang et al., 2016). Aqueous film‐forming foams are essential to effectively suppress fires associated with hydrocarbon‐based fuels, including gasoline and jet fuel, and therefore, are widely used at airports, military bases, chemical plants, and other fire training areas. Although AFFFs are critical to protecting human life and property, the release of their component PFAS to water and uptake by biota are of concern (Ankley et al., 2021).

Several PFAS are frequently detected in the water and soil near sites where AFFFs have been applied (Anderson et al., 2016; East et al., 2021). Although perfluorooctane sulfonate (PFOS) tends to be present at the highest concentrations, other PFAS (e.g., perfluorohexane sulfonate [PFHxS] and perfluorooctanoic acid [PFOA]), as well as PFAS precursors (e.g., 6:2 fluorotelomer sulfonate [6:2 FTS]), can contribute substantially to environmental exposure (East et al., 2021). In the present study, mean concentrations of PFOS, PFHxS, PFOA, and 6:2 FTS in surface waters near Air Force bases were found to be 0.25, 0.23, 0.07, and 0.09 µg/L, respectively; however, it should be noted that variation was extensive within and across sites. For example, maximum surface water concentrations detected in a similar data set indicate that aquatic life can be exposed to PFAS concentrations an order of magnitude greater than the reported mean levels (up to ~9.0 µg/L PFOS; Anderson et al., 2016).

Despite the prevalence of PFAS in aquatic habitats, their toxicity is not well characterized for most wildlife due to a lack of or limited toxicity data available for many relevant taxa including amphibians. Although basic PFAS ecotoxicological data are lacking for many amphibians, evidence is accumulating that PFAS can have adverse effects on this group. Indeed, PFAS exposure has been associated with altered growth, development, and body condition in larval and terrestrial forms of amphibians (Ankley et al., 2004; Flynn et al., 2019, 2020, 2021; Hoover et al., 2017). Furthermore, PFAS exposure has been linked to altered thyroid function (Cheng et al., 2011; Melzer et al., 2010). Given their thyroid‐mediated development, amphibians may be especially susceptible to PFAS during early aquatic development (Tata, 2006). However, because chronic toxicity of PFAS has been evaluated in only a few (six species total) amphibian species (Ankley et al., 2021), sensitivities of different amphibian taxa to PFAS remain poorly known. To address this knowledge gap, we designed our study to characterize the effects of chronic PFAS exposure on survival, growth, and development in three species of larval amphibians: northern leopard frogs (*Rana [Lithobates] pipiens*), American toads (*Anaxyrus americanus*), and eastern tiger salamanders (*Ambystoma tigrinum*). Our secondary aim was to assess how aquatic PFAS exposure could affect thyroid form and function in toads and salamanders. We hypothesized that sensitivity would be driven by duration of larval stages, with American toads being the least sensitive due to their rapid developmental rate, followed by northern leopard frogs, and eastern tiger salamanders.

## MATERIALS AND METHODS

### Animal collection and husbandry

The present study was conducted under the auspices of the Purdue University Institutional Animal Care and Use Committee (protocol 1601001355). Egg masses of northern leopard frogs (*n* = 13), eastern tiger salamanders (*n* = 63), and American toads (*n* = 15) were collected from an ephemeral pond with no history of PFAS inputs in West Lafayette, Indiana (USA) in March 2018, February 2017, and May 2017, respectively. We selected these three species for several reasons: (1) their broad distribution and high abundance across the United States means they are likely to occur at sites affected by PFAS (Petranka, 1998; Werner et al., 2007); (2) these species represent some of the most diverse amphibian families in North America (Ranidae, Bufonidae, and Ambystomatidae); and (3) these species have become common ecotoxicology models due to the aforementioned factors and their ease of husbandry for laboratory studies (Boone & Bridges, 2003).

Frog and toad egg masses were transferred to separate 100‐L outdoor wading pools. After hatching, larvae were fed Purina® Rabbit Chow ad libitum, and water changes were done as needed. After reaching Gosner stage 25 (Gosner, 1960), we haphazardly distributed groups of 20 larvae to 15‐L polypropylene tubs to acclimate to laboratory conditions (20 ± 2 °C, 12:12‐h light:dark cycle) for 1 week prior to the start of the experiments. Larvae with visible irregularities in morphology, coloration, or behavior were excluded. In the laboratory, all larvae were fed ground Tetramin® flakes ad libitum, and 100% water changes were performed twice weekly until the start of the experiments.

Due to differences in life histories, salamander husbandry differed from that of the other two species. Each salamander egg mass was assigned to a 15‐L polypropylene tub in the laboratory. After hatching, larvae were fed zooplankton or blackworms daily ad libitum, and water changes were done as needed, until all individuals had reached Harrison stage 46 (Harrison, 1969). One week prior to the start of the experiment, larvae were transferred to individual polypropylene 1‐L cups for acclimation. Larvae with visible irregularities in morphology, coloration, or behavior were excluded.

### Chemicals and stock solution preparation

We purchased PFOA (SKU‐Pack Size #171468‐25G, 96% or more pure), PFOS (SKU‐Pack Size #77282‐10G, 98% or more pure), and PFHxS (SKU‐Pack Size #50929‐10G‐F, 98% or more pure) from Sigma‐Aldrich and 6:2 FTS (SKU‐Pack Size #6164‐3‐06, 98% or more pure) from SynQuest Laboratories. We prepared primary stock solutions (200–1000 mg/L) by dissolving chemicals into 1 L of ultraviolet (UV)‐irradiated filtered well water. We mixed the solutions on a stir plate until the chemicals dissolved (2 h for PFOA and PFHxS, and 8 h for PFOS and 6:2 FTS). The 6:2 FTS solution was further vacuum‐filtered to remove any remaining insoluble solids (ashless, grade 40; Whatman). Stock solutions were stored in polypropylene bottles and refrigerated until used to make test solutions.

### Experimental design

Our study design included a no‐PFAS control and a factorial combination of four PFAS (PFOA, PFOS, PFHxS, or 6:2 FTS) crossed with three concentrations (10, 100, or 1000 μg/L). For all species, our experimental units were 15‐L tubs; however, because of the cannibalistic nature of salamanders, they were kept in individual 1‐L cups inside the tubs. For frogs and toads, tubs were filled with 7.5 L of filtered, UV‐irradiated well water and assigned 20 individuals to each tub. For salamanders, cups were filled with 750 ml of filtered, UV‐irradiated well water and assigned one individual per cup, for a total of 15 cups/tub. Each treatment was replicated 4 times for a total of 52 experimental units/species. Experimental units were arranged in a randomized block design with shelf height as the blocking factor (*n* = 4 blocks) and replicates of each treatment equally represented in each block. Animals were maintained at 20 ± 2 °C with a 12:12‐h light:dark photoperiod for the duration of the exposure. Water changes were conducted weekly for salamanders and twice weekly for frogs and toads. Larvae were fed (toad tadpoles for salamanders, ground Tetramin® for frogs and toads) at 20% of the average larval body weight every other day (i.e., 10% of mean body wt daily). We determined feeding rations using extra‐experimental units and animals (*n* = 4 bins/species) that were independent of the experiment (i.e., not included in any analyses) but were set up identically to the control treatments of each species. These animals were weighed weekly to calculate food rations.

Larvae were exposed using a static renewal approach with all water removed and replaced with freshly spiked PFAS‐contaminated water at the scheduled water changes, which were conducted every 4 days. Animals were monitored daily for mortality and abnormalities. We collected water samples (~5 ml) immediately after initial exposure and immediately prior to the first water change for verification of PFAS concentrations. A previous study in this laboratory showed that our methods resulted in consistent PFAS exposure concentrations to animals throughout a 30‐day experiment (Hoover et al., 2017).

We sampled larvae to quantify snout–vent length (SVL), body mass, and developmental stage (frogs and toads only). The SVL and body mass were used to calculate the scaled mass index (SMI) as described in *Data analysis and statistics*. Frog and salamander larvae were sampled after 30‐day exposures. Because toads began metamorphosing on Day 26, we opted to continue exposures through metamorphosis to standardize developmental stage and ensure that morphological data collected were more directly comparable across treatments. Therefore, we used days to metamorphosis (rather than developmental stage) as the developmental endpoint for toads collected at the final sampling point.

We also preserved the heads of five toads and five salamanders from each experimental unit in 10% buffered formalin for thyroid histopathology. The remaining animals were stored whole at −80 °C for thyroid hormone quantification. Because there were no substantial effects of PFAS on these response variables, the methods and detailed results for thyroid histology and hormone concentrations are presented in the Supporting Information.

### Water PFAS quantification

The methods used for quantification of PFAS in test water are described in detail in previous publications and their associated supplementary information (Abercrombie et al., 2019; Hoover et al., 2017). Briefly, extracts were analyzed using reverse‐phase liquid chromatography mass spectrometry with either an AB Sciex Quadrupole Time‐of‐Flight (QToF) 5600 or an AB Sciex 3000 triple quadrupole mass spectrometer.

### Data analysis and statistics

All data analysis was carried out in R (R Ver 3.5.3; R Core Team, 2019). All response variables were log_10_‐transformed prior to analysis to better approximate normal distributions. Log‐transformed data and model residual distributions were inspected visually for normality and skewness. Values for PFAS detected in water that were less than the lower limit of quantitation (LLOQ; Supporting Information, Table S1) were included as one‐half the LLOQ. Values that were less than the detection limit (Supporting Information, Table S1) were reported as 0.

Analyses of morphological (SVL, mass, and body condition) and developmental (Gosner stage and time‐to‐metamorphosis) endpoints were conducted using linear mixed models. We calculated SMI as a measure of body condition and lipid reserves using the approach of Peig and Green (2009):

$$\hat{M_{i}}=M_{i}\;{\left[\frac{L_{0}}{L_{i}}\right]}^{b_{\text{SMA}}}$$

where $\hat{M_{i}}$ is the estimated body mass of individual *i* when the SVL is standardized to the mean SVL of the data set (*L*_0_). The body mass and SVL of individual *i* are given by *M*_*i*_ and *L*_*i*_, respectively. Finally, *b*_SMA_ is defined as the allometric scaling exponent of the standardized major axis regression of log_10_‐mass on log_10_‐SVL of salamanders from the control treatment. We calculated scaling coefficients for SMI estimation using only animals from controls for each species, at each time point to ensure coefficients were not skewed by treatment effects.

In all models, chemical treatment was included as a fixed predictor with 13 levels, and experimental unit was used as a random effect to account for the nonindependence of larvae sharing experimental units. Salamanders were reared in “individual” containers nested within experimental units. The effect of a treatment was considered significant if that treatment was associated with a *p* < 0.05, indicating a statistical difference from the control group.

## RESULTS

### Measured PFAS in test media

Measured PFAS levels across treatment concentrations within a chemical were well separated and differed, as intended, by roughly a factor of 10 (Table 1). Mean PFAS levels in water from controls varied slightly across species, but were always less than 1 μg/L (see Table 1 for details). Furthermore, all control water samples tested were less than the LLOQ, except for PFOA in toads (81% greater than the LLOQ).

**Table 1:** Summary of mean measured water per‐ and polyfluoroalkyl substance concentrations by nominal treatment concentration for *Rana pipiens* (northern leopard frog), *Anaxyrus americanus* (American toad), and *Ambystoma tigrinum* (eastern tiger salamander)^a^

		(Measured concentration (SD))		
Target PFAS	Nominal concentration	*R. pipiens*	*A. americanus*	*A. tigrinum*
PFOA	0	0.1 (0)	0.37 (0.21)	0.05 (0.04)
	10	9.64 (0.88)	10 (0.77)	11.05 (1.2)
	100	125.1 (7.25)	94.03 (11.12)	101.49 (7.5)
	1000	1376.19 (130.44)	872.75 (72.43)	867.73 (74.95)
PFOS	0	0.12 (0)	0 (0)	0 (0)
	10	7.74 (1.44)	5.1 (1.15)	4.36 (0.56)
	100	121.89 (13.58)	54.55 (4.22)	55.21 (4.75)
	1000	1436.78 (99.03)	616.18 (53.37)	621.27 (61.08)
PFHxS	0	0.16 (0.11)	0 (0.01)	0.01 (0.01)
	10	9.57 (1.15)	7.2 (0.84)	7.33 (0.94)
	100	119.54 (7.91)	80.6 (7.42)	80.19 (6.48)
	1000	1306.64 (90.4)	634.44 (345.89)	796.7 (71.82)
6:2 FTS	0	0.1 (0)	0 (0.01)	0 (0.01)
	10	12.59 (0.99)	8.05 (0.58)	7.83 (0.75)
	100	175.26 (17.11)	82.34 (6.44)	108.57 (45.74)
	1000	1795.28 (77.62)	782.65 (106.04)	858.83 (115.59)

^a^ All concentrations are reported in µg/L.

FTS = fluorotelomer sulfonate; PFAS = per‐ and polyfluoroalkyl substances; PFOA = perfluorooctanoic acid; PFHxS = perfluorohexane sulfonate; PFOS = perfluorooctane sulfonate.

### Effects of chronic PFAS exposure on survival

Across all species and treatments, mean survival was more than 85% for the duration of the studies. Leopard frog survival was greater than 93% in every treatment and did not differ among treatments (*F*_12,39_ = 0.91, *p* = 0.55). Overall salamander survival was 99%. Although we did not observe an overall effect of treatment on salamander survival (*F*_12,39_ = 1.55, *p* = 0.15), survival in the 6:2 FTS 100‐µg/L treatment (96%) was reduced relative to the control (*t* = −2.79, *p* = 0.008). Survival in toads was the lowest of the three species (86%), but was still high and did not differ among treatments (*F*_12,39_ = 1.4, *p* = 0.21).

### Effects of chronic PFAS exposure on morphology and development

**Figure 1:**
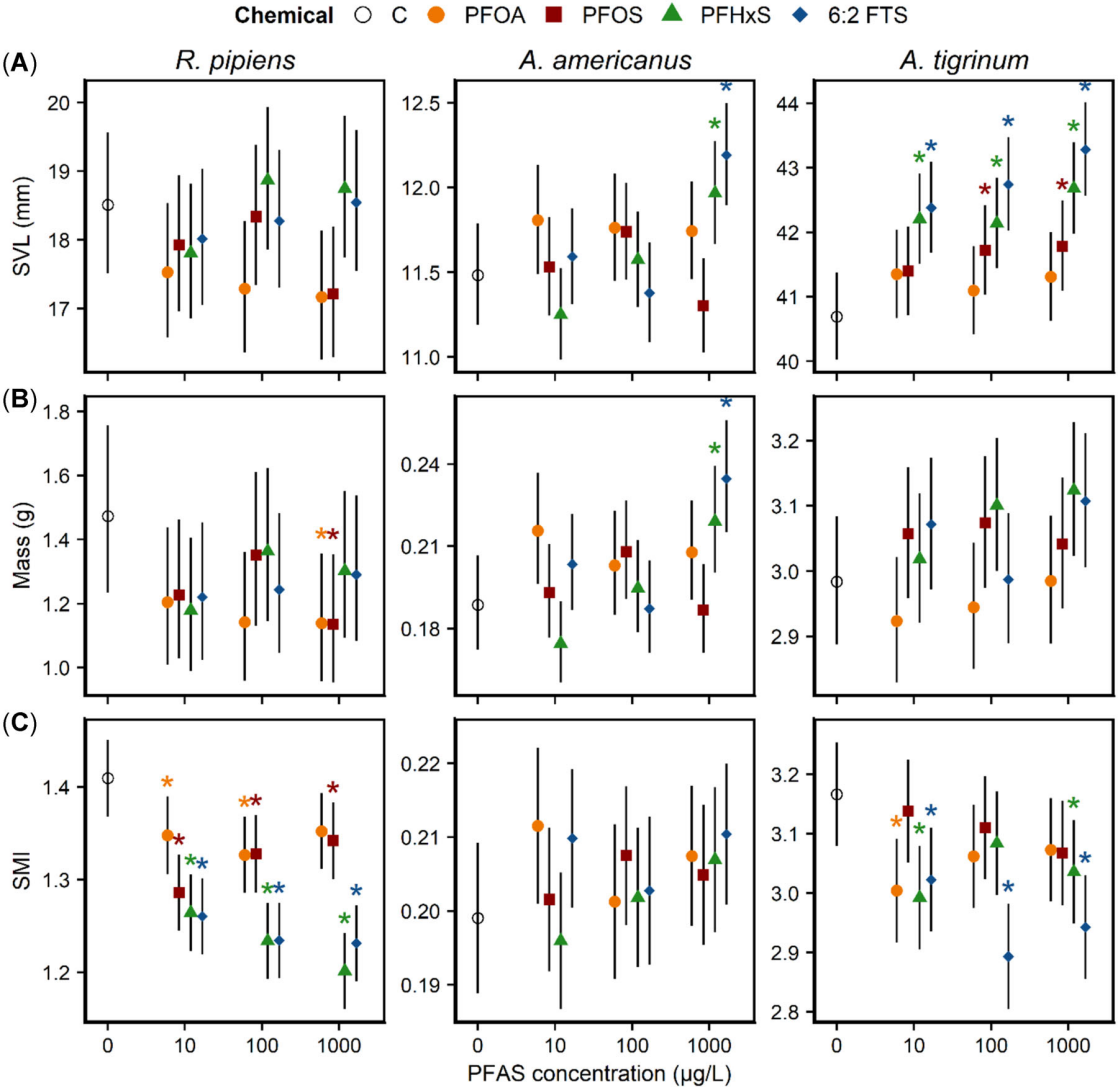
Model‐derived means (95% confidence interval) associated with (**A**) snout–vent length, (**B**) body mass, and (**C**) body condition (scaled mass index [SMI]) at the conclusion of per‐ and polyfluoroalkyl substances (PFAS) exposure studies for *Rana [Lithobates] pipiens* (northern leopard frogs; 30 days), *Anaxyrus americanus* (American toads; metamorphosis, 26–45 days), and *Ambystoma tigrinum* (eastern tiger salamanders; 30 days). Asterisks denote significant differences of a treatment from the control (*p* < 0.05). C = control; PFOA = perfluorooctanoic acid; PFOS = perfluorooctane sulfonate; PFHxS = perfluorohexane sulfonate; 6:2 FTS = 6:2 fluorotelomer sulfonate.

The effects of PFAS on morphological endpoints varied among species, PFAS, and endpoints. Per‐ and polyfluoroalkyl substance exposure resulted in increased SVL only in toads and salamanders (Figure 1A). Toad SVL was only affected by the 1000‐µg/L treatments of PFHxS and 6:2 FTS (~5% larger than controls). Relative to the controls, salamander SVL was slightly elevated in all concentrations of PFHxS and 6:2 FTS, and in the 100‐ and 1000‐μg/L PFOS treatments (~3%–6%). Body mass was the least sensitive and most inconsistent phenotypic endpoint examined (Figure 1B). However, when effects were present, they were relatively large. For example, frog body mass was reduced by PFOA and PFOS at 1000 μg/L (~30%) and elevated in toads for PFHxS and 6:2 FTS at 1000 μg/L (10%–15%) relative to controls. The mass of salamander larvae was not obviously affected by any PFAS treatment, although in general body mass means were greater in PFAS treatments than controls.

Per‐ and polyfluoroalkyl substance treatment generally reduced body condition (SMI) by Day 30, but variation was substantial among species (Figure 1C). Frog SMI was reduced in every PFAS treatment. Relative to the control, reductions in frog body conditions were similar between PFOA and PFOS (~5%–10%) and PFHxS and 6:2 FTS (~10%–17%). Salamander SMI was reduced in all 6:2 FTS treatments (~5%–10%), in PFHxS treatments at 10 and 1000 µg/L (~5%), and in PFOA treatments at 10 µg/L (~5%). Toad body condition was more variable compared with the other two species, and we did not detect an effect of any of the PFAS tested.

**Figure 2:**
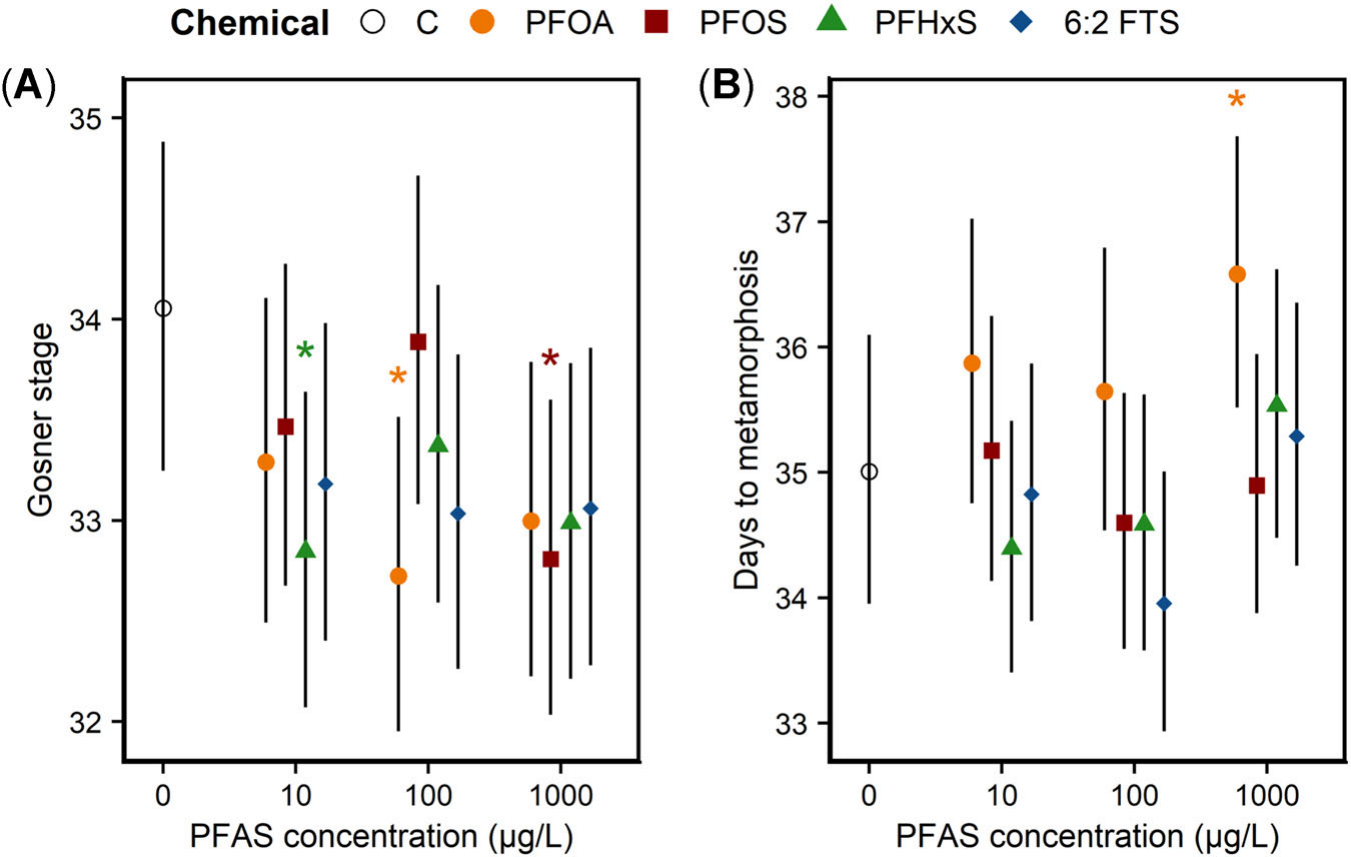
Model‐derived means (95% confidence interval) associated with (**A**) developmental stage of northern leopard frogs after 30 days of exposure to per‐ and polyfluoroalkyl substances (PFAS) and (**B**) days required for American toads to reach metamorphosis. Asterisks denote significant differences of a treatment from the control (*p* < 0.05). C = control; PFOA = perfluorooctanoic acid; PFOS = perfluorooctane sulfonate; PFHxS = perfluorohexane sulfonate; 6:2 FTS = 6:2 fluorotelomer sulfonate.

The effects of PFAS on larval development differed between species (Figure 2). Frog development was delayed by approximately 1 Gosner stage in the 10‐µg/L PFHxS, 100‐µg/L PFOS, and 1000‐µg/L PFOS treatments (Figure 2A). In contrast, for toads, time to metamorphosis was only affected by 1000‐µg/L PFOA, which delayed metamorphosis by approximately 2 days (Figure 2B).

**Figure 3:**
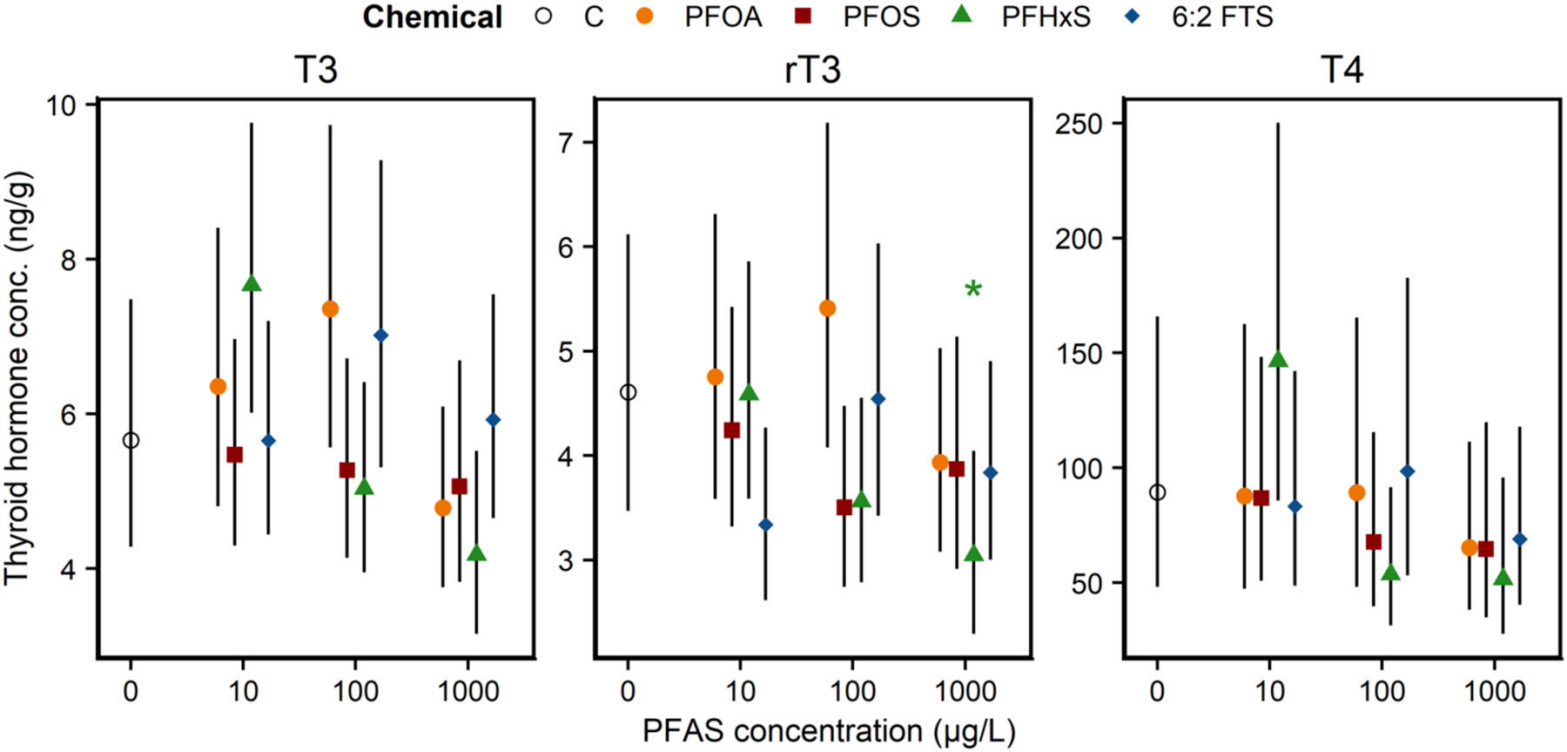
Mean concentrations (95% confidence interval) of triiodothyronine (T3), thyroxine (T4), and reverse T3 (rT3) in whole‐body homogenates of American toads at the climax of metamorphosis. Overall, evidence was scant that per‐ and polyfluoroalkyl substance (PFAS) exposure affected thyroid hormone levels in toads, with the exception of the 1000‐µg/L perfluorohexane sulfonate (PFHxS) treatment, which reduced mean rT3 levels by 27% relative to the control. C = control; PFOA = perfluorooctanoic acid; PFOS = perfluorooctane sulfonate; 6:2 FTS = 6:2 fluorotelomer sulfonate.

Thyroid hormone levels and gland development, at the developmental stages examined, were only appreciable in toads (Supporting Information, Figures S1 and S2). For toads, no effects of PFAS exposure were noted on thyroid gland histology (Supporting Information, Figure S2). There was also little evidence that these PFAS treatments affected whole‐body thyroid hormone levels, although the 1000‐µg/L PFHxS treatment was associated with a statistically significant decrease in reverse triiodothyronine (rT3) levels (Figure 3).

## DISCUSSION

We found that chronic PFAS exposure affected the size, body condition, and development of northern leopard frog, American toad, and eastern tiger salamander larvae. However, the extent of PFAS effects on amphibian larvae was highly dependent on the species and chemical being tested. In general, toads appeared to be less sensitive to PFAS exposure than either frogs or salamanders. Furthermore, SMI was the most sensitive endpoint associated with PFAS exposure and was consistently reduced at the lowest exposure concentrations tested, with lowest‐observed‐effect concentration (LOEC) values of 10 µg/L for all PFAS in frogs and of 10 µg/L for salamanders for all PFAS except PFOS. Although the 100‐ and 1000‐µg/L treatment levels are not reflective of PFAS concentrations likely to be encountered by wildlife, PFAS can approach 10 µg/L in surface waters at heavily affected sites (Anderson et al., 2016). This indicates that amphibian growth and development could be altered by PFAS exposure at such sites. We note that water concentrations of PFAS in the 100‐ and 1000‐µg/L treatments for frogs were at times up to 2× greater than those for salamanders and toads. This means that caution is warranted when one is directly comparing the effects observed on frogs in those treatments with those for the other species. However, measured PFAS concentrations in the 10‐µg/L treatments were consistent across species, so direct comparison of LOECs between species remains straightforward.

Although toad body condition was not affected by treatments, PFAS reduced the body condition of frogs and salamanders. Frog body condition was reduced by all PFAS‐concentration treatments tested, with the largest reductions coming from PFHxS and 6:2 FTS. Salamander body condition was reduced by every PFAS except for PFOS, and the greatest reductions in body condition were associated with exposure to 6:2 FTS. Body condition, which integrates both animal size and mass, has the potential to provide greater insight into the effects of PFAS than either measure alone. For example, although the body condition of frogs and salamanders were both reduced by PFAS exposure, the underlying morphological changes differed between species, which indicates that physiological responses to PFAS could differ among taxa. In frogs, PFAS exposure did not alter SVL in a consistent way, but treatments tended to reduce body mass. Alternatively, salamander SVL was consistently greater in PFAS treatments, whereas mass remained relatively unchanged. Thus the body condition metric was able to detect a consistent response to PFAS exposure that would not be readily apparent from the SVL and mass data alone. Given that body condition can be positively associated with adult survival in amphibians (Reading, 2007), the measure is likely to be relevant to the health of wild populations. The mechanism(s) underlying these morphological changes are unknown, but PFAS‐induced alterations to lipid metabolism could be playing a role, because studies increasingly show that PFAS can affect lipid metabolism through several mechanisms and signaling pathways (see Jiang et al., 2015; Seyoum et al., 2020). Although we did not directly examine energy reserves, the consistent effects of PFAS on amphibian body condition indicate that exposure has the potential to alter the nature of how amphibians grow in early development.

Although few studies have examined the effects of chronic PFAS exposure on larval amphibians, those that do exist offer useful comparisons with our results. To our knowledge, four other studies have tested how aquatic chronic (more than 30 days) exposure to PFOS can affect amphibian larvae. One study examined how development, thyroid morphology, and gene expression in *Xenopus laevis* larvae were affected by exposure to 0.1–100 µg/L of PFOS, but did not measure size or body condition (Cheng et al., 2011). They found limited evidence of PFOS delaying development and saw no effects on thyroid histology. However, they consistently observed nonmonotonic expression of developmental genes in response to PFOS. Two other studies examined chronic PFOS exposure in northern leopard frog larvae (Ankley et al., 2004; Hoover et al., 2017). Ankley et al. (2004) found no consistent effects of PFOS exposure on tadpole mass or length, but did see evidence that PFOS has the potential to alter larval development at concentrations as low as 30 ppb. In addition to PFOS, Hoover et al. (2017) assessed the effects of PFOA, PFHxS, and 6:2 FTS on tadpole size and development. They found that PFOS and PFHxS delayed larval development after 40 days of exposure to 100 and 10 µg/L, respectively. There was limited evidence that PFAS affected larval growth (i.e., *p* = 0.058), although mean SVL tended to be reduced in tadpoles exposed to PFOS, PFHxS, and 6:2 FTS. The final study assessed the toxicity of PFOA and PFOS in simulated pond communities (i.e., mesocosms), in which tanks were dosed with PFAS‐spiked sediment, which allowed PFAS to equilibrate between water and sediment more naturally and become incorporated into the diet (i.e., algae; Flynn et al., 2020). Although less controlled than the aforementioned laboratory studies, the authors found that PFOS delayed development at surface water concentrations as low as 0.60 µg/L. Although more studies would be needed to determine whether these results are generalizable, they indicate that toxicity studies in which organisms are exposed only via water could underestimate PFAS toxicity in the environment. Overall, the PFAS‐associated alterations in the size and body condition of larval amphibians in our study are generally consistent with those found in the few other comparable studies available. Together, these results offer further evidence that exposure to PFAS at highly affected sites could affect larval growth or development.

The distinct responses among the three species offer insights into how PFAS can differentially affect amphibian taxa. Taking our data along with those of previous research, we were able to compare the effects of aquatic, dietary, and dermal PFAS exposure in eastern tiger salamanders. Dietary and dermal exposure were also associated with reduced body conditions driven by increases in SVL coupled with no changes in mass in salamanders (Abercrombie et al., 2021; Flynn et al., 2021). This pattern indicates that PFAS could interfere with lipid metabolism or other physiological processes in a way that results in larger animals with fewer energy reserves. Similarly, the absence of effects on toad body condition is consistent with the results of a dermal exposure study in metamorphic toads (Abercrombie et al., 2021). The effects of PFAS on northern leopard frog body condition appear to be less consistent. Metamorphic northern leopard frogs dermally exposed to PFAS had higher body conditions than controls, contrary to the results of the present study (Abercrombie et al., 2021). Together, these data indicate several research areas that could be explored to understand species‐level variation in amphibian PFAS toxicity. Based on our data, amphibians with longer larval periods and/or slower life histories (e.g., ranids and some ambystomids) could be more sensitive to PFAS than those with shorter larval periods and/or faster life histories (e.g., bufonids). Toads are capable of using bodies of water with much shorter and more variable hydroperiods than frogs and salamanders (Lannoo, 2005) due to their ability to accelerate development to complete metamorphosis rapidly. Frogs and salamanders often need 3+ months to complete aquatic development, whereas toads only require 1–1.5 months (Lannoo, 2005). Given the role of thyroid function on amphibian development and the potential for PFAS to disrupt thyroid signaling, these species‐level differences in PFAS susceptibility may be mediated in part by differences in the regulation of thyroid hormones among species. In amphibians and other taxa with thyroid‐mediated development, we would also expect PFAS to disrupt larval development. Although development was more variable than body condition in our study, the effects of PFAS on development in frogs and toads mirrored the effects on body condition. Per‐ and polyfluoroalkyl substance exposure was associated with minor developmental delays in leopard frogs. Furthermore, evidence was scant that PFAS negatively affected toad development, with only the highest PFOA treatment being associated with delayed metamorphosis.

Even though PFAS altered development in toads, there was limited evidence that exposure affected thyroid gland structure or thyroid hormones (i.e., T3, rT3, and T4). We note that the lack of substantial effects on thyroid endpoints in our study could be due to the small sample sizes, analytical methods, range of treatment concentrations, or other aspects of our methodology. The thyroid gland histology showed rapidly growing and developing tissue in both treatment and control salamanders. No significant differences were observed because the glands were variable in size, the follicles were consistently small, and the follicular cells were pleomorphic. Mature, colloid‐producing glands were found in toads, but again, no treatment amount influenced the histological appearance of the gland. This is further supported by the hormone data, with no treatment group significantly deviating from control animals for any of the three hormones examined. Given the lack of morphological differences between these groups, it perhaps follows that the thyroid hormone levels and the thyroid glands themselves did not show significant changes. Those species that did show toxic effects (e.g., frogs) may indeed show alterations to the thyroid gland once the gland has had more of a chance to develop (i.e., nearing metamorphosis).

## CONCLUSIONS

Our study offers important insights into the relative sensitivity of three common North American amphibian species to common PFAS. We determined that chronic PFAS exposure can negatively affect larval amphibian growth and development. However, the observed effects were highly species dependent. Our results indicate that evaluating the toxicity of PFAS using a single species is not sufficient for protective amphibian risk assessment. Future studies assessing the effects of PFAS on amphibians would be needed to determine whether these differences in susceptibility can be predicted from species' life histories (e.g., slow vs. rapid aquatic development). Given that amphibians already face substantial declines around the world (International Union for the Conservation of Nature, 2021), the potential for these ubiquitous environmental stressors to negatively affect amphibian growth and development is concerning. The potential for amphibians with lengthy larval periods to be adversely affected warrants consideration when managing for the long‐term survival of these species. Future studies could focus on these species and perhaps lengthen the exposure period so that developmental endpoints that are best measured near or at metamorphosis (including thyroid development) may be fully examined. Finally, consideration of finer scale endpoints, including expression of lipid and thyroid‐responsive genes, would be beneficial to further elucidate the mechanisms of toxicity.

## Supporting Information

The Supporting Information is available on the Wiley Online Library at https://doi.org/10.1002/etc.5319.

## Disclaimer

Any use of trade, firm, or product names is for descriptive purposes only and does not imply endorsement by the authors or the US Government.

## Supporting information

This article includes online‐only Supporting Information.

Supporting information.

## Data Availability

Data are available through the Purdue University Research Repository (https://purr.purdue.edu/publications/3980/1). Data, associated metadata, and calculation tools are also available from the corresponding author (mssepulv@purdue.edu).
