# Supplementary material for: Comparative Toxicity of Aquatic Per‐ and Polyfluoroalkyl Substance Exposure in Three Species of Amphibians
Source: Environ Toxicol Chem. 2022 Mar 31;41(6):1407–15. doi: 10.1002/etc.5319 (PMC9314107; doi:10.1002/etc.5319)
Supplement: Supplementary file 1 — Supporting information. [file ETC-41-1407-s001.docx]

**Supplemental Materials**

*Thyroid histology methods*

For salamanders, the head and cervical region of five animals per group were taken and preserved in 10% NBF. After sufficient time (>48 h) to fix the tissue, three heads per experimental unit were bisected sagittally along the midline and the right halves of the heads were placed in histology cassettes. Heads were decalcified in 14% EDTA for 1 week, embedded in paraffin and sectioned starting 400 µm into the tissue and a minimum of 6 sections were taken 50 µm apart. Sections were set two per slide and stained with routine hematoxylin and eosin (H&E) staining.

For toads, the head and cervical region of at least 6 animals per experimental unit were taken and preserved in 10% NBF. After sufficient time (>48 h) for fixation, 6-7 heads per experimental unit were embedded whole in paraffin blocks, oriented with the rostrum deep in the block, allowing for initial sectioning of the cervical tissue in a transverse plane. Blocks were cut down until thyroid glands were visible from at least 1-2 heads per block, then sectioned at a thickness of 6 µm. Six sections 30-50 µm apart were set two per slide and stained with routine H&E staining. Initially, only the high dose (1000 μg/L) treatments were examined. Differences in follicular cell height based on chemical treatment were not statistically significant, but due to a data trend suggesting an effect of chemical, the low doses (10 μg/L) of PFOS and PFHxS were also examined.

*Thyroid hormone concentrations methods*

Hormones quantified included triiodothyronine (T3), thyroxine (T4) and reverse T3 (rT3). Reverse triiodothyronine is believed to be metabolically inactive and differs from (T3) in the positions of the iodine atoms attached to the aromatic rings with the majority being formed by peripheral deiodination of T4 (Shivaraj et al., 2009). Whole body homogenates were used as blood was difficult if not impossible to sample from these very small animals. Hormone measurements were only available for toads, as salamanders did not achieve enough development of the thyroid gland to allow for measurable levels of hormone in whole body homogenates (data not shown). Whole bodies were placed in a ceramic mortar and frozen with liquid nitrogen, then ground into a fine powder. This sample was then extracted and analyzed using a previously reported method (Bussy et al., 2017). Briefly, approximately 200 mg of each sample was mixed with PBS to achieve a concentration of 1 g wet tissue/mL. Samples were spiked with the internal standard solution (1000 pg/mL final concentration in sample). After being vortexed, samples were incubated at 4 ºC away from the light for one hour. Enzymatic digestion was performed using 100 uL of buffer and pronase enzyme, which was used at the concentration of 4 units per mg wet tissue. Samples were then incubated overnight at 37 ºC away from light. Sample clean-up was achieved by protein precipitation by mixing the samples with 1 mL MeOH and incubated away from light for 30 min in a water/ice bath. The samples were then centrifuged at 15,000 × g for 10 min and the supernatant was transferred to a new tube and evaporated in vacuo. The dry residue was reconstituted in 1 mL MeOH/H2O (1:1) mixture and kept at -20 ºC until analysis by LC-MS/MS. A Waters Xevo TQ-S triple quadrupole mass spectrometer was used for the detection and quantification of thyroid hormones. Chromatographic separation was achieved by an H-Class UPLC system with a Waters BEH C18 column (2.1 × 100 mm, 1.7 um particle size). Solvents A and B were water (0.1% formic acid) and methanol, respectively. The flow rate was kept at 0.2 mL/min and the following gradient was applied (time in minutes; % of A): (initial; 50), (5; 60), (8; 50), (10; 10), (11; 1), (12; 1), (12.01; 82), and (14; 50). Data were acquired with MassLynx 4.1 and processed for calibration and quantification of the analytes with TargetLynx software (Waters).

*Thyroid histology and hormone concentration results*

The thyroid glands of larval salamanders were early in their development and histological findings are consistent with rapidly growing tissue (**Figure S1**). The largest follicles in each section consisted of a narrow lumen and bordered by no more than 15-20 follicular epithelial cells. These follicular cells contained large nuclei with loose, open chromatin, a high nuclear: cytoplasm ratio, and multiple nucleoli visible. Occasional mitotic figures and little to no colloid was present within the follicle lumina. No treatment-related differences were observed.

Toad thyroid glands were much more developed, with cuboidal epithelial cells lining colloid-containing lumina (**Figure S2**). No significant changes were seen on histopathology, but follicular cell height was slightly higher on average (though not statistically significant) in PFOS- (3.23 ± 0.23 µm) and PFHxS-treated (3.21 ± 0.24 µm) animals when compared to controls (2.94 ± 0.15 µm). In addition, T3 and T4 thyroid hormone levels in toad metamorphs (whole body homogenates) did not differ among treatments (p > 0.05) (**Figure S3**). However, rT3 levels in toads exposed to 1000 ng/L PFHxS were reduced by 27% relative to the control. The results of the thyroid hormone analysis should be interpreted cautiously, as the combination of variation in thyroid hormone levels within treatments, coupled with limited samples sizes (n = 3-4) severely limited our power to detect differences among treatments. Means (and ranges) for T3, rT3 and T4 were and 5.90 (3.11 – 9.85), 4.15 (2.29 – 7.39), and 87.85 (16.66 – 179) ng/g, respectively.

| **Table S1**. Summary of mean measured water concentrations by species, chemical, and nominal concentration. All concentrations are reported in µg/L. | | | | |
| --- | --- | --- | --- | --- |
|  |  |  | | |
|  |  | Frog | Toad | Salamander |
| Target PFAS | Nominal concentration | Measured concentration (SD) | | |
| PFOA | 0 | 0.1 (0) | 0.37 (0.21) | 0.05 (0.04) |
|  | 10 | 9.64 (0.88) | 10 (0.77) | 11.05 (1.2) |
|  | 100 | 125.1 (7.25) | 94.03 (11.12) | 101.49 (7.5) |
|  | 1000 | 1376.19 (130.44) | 872.75 (72.43) | 867.73 (74.95) |
| PFOS | 0 | 0.12 (0) | 0 (0) | 0 (0) |
|  | 10 | 7.74 (1.44) | 5.1 (1.15) | 4.36 (0.56) |
|  | 100 | 121.89 (13.58) | 54.55 (4.22) | 55.21 (4.75) |
|  | 1000 | 1436.78 (99.03) | 616.18 (53.37) | 621.27 (61.08) |
| PFHxS | 0 | 0.16 (0.11) | 0 (0.01) | 0.01 (0.01) |
|  | 10 | 9.57 (1.15) | 7.2 (0.84) | 7.33 (0.94) |
|  | 100 | 119.54 (7.91) | 80.6 (7.42) | 80.19 (6.48) |
|  | 1000 | 1306.64 (90.4) | 634.44 (345.89) | 796.7 (71.82) |
| 6:2 FTS | 0 | 0.1 (0) | 0 (0.01) | 0 (0.01) |
|  | 10 | 12.59 (0.99) | 8.05 (0.58) | 7.83 (0.75) |
|  | 100 | 175.26 (17.11) | 82.34 (6.44) | 108.57 (45.74) |
|  | 1000 | 1795.28 (77.62) | 782.65 (106.04) | 858.83 (115.59) |


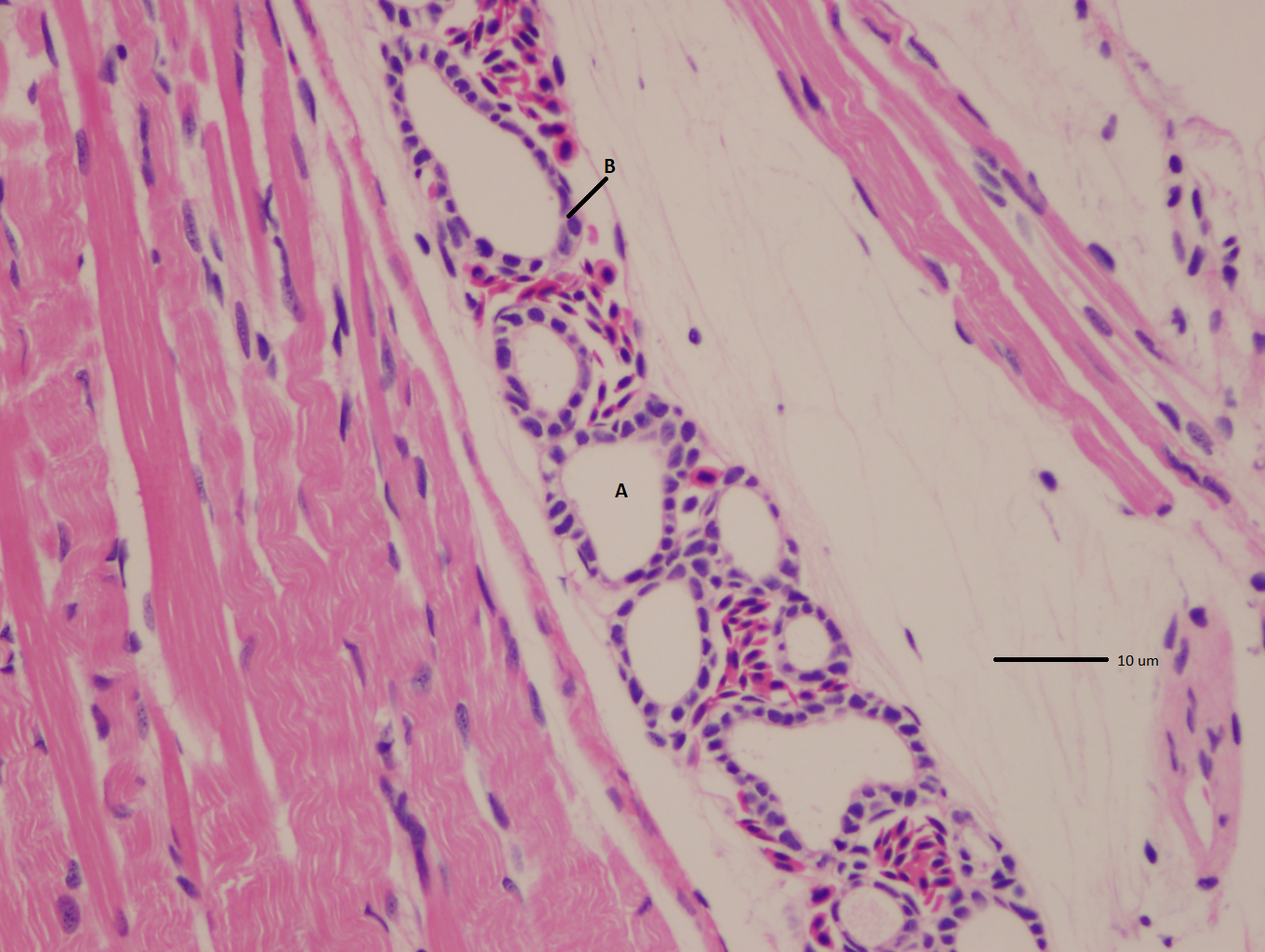


**Figure S1**. Salamander thyroid photomicrograph. Elongated gland shape can be appreciated in sagittal section. (A) Small follicular size with limited colloid within follicles. (B) Thyroid epithelium showing high N:C ratio, pleomorphism, and prominent nucleoli.


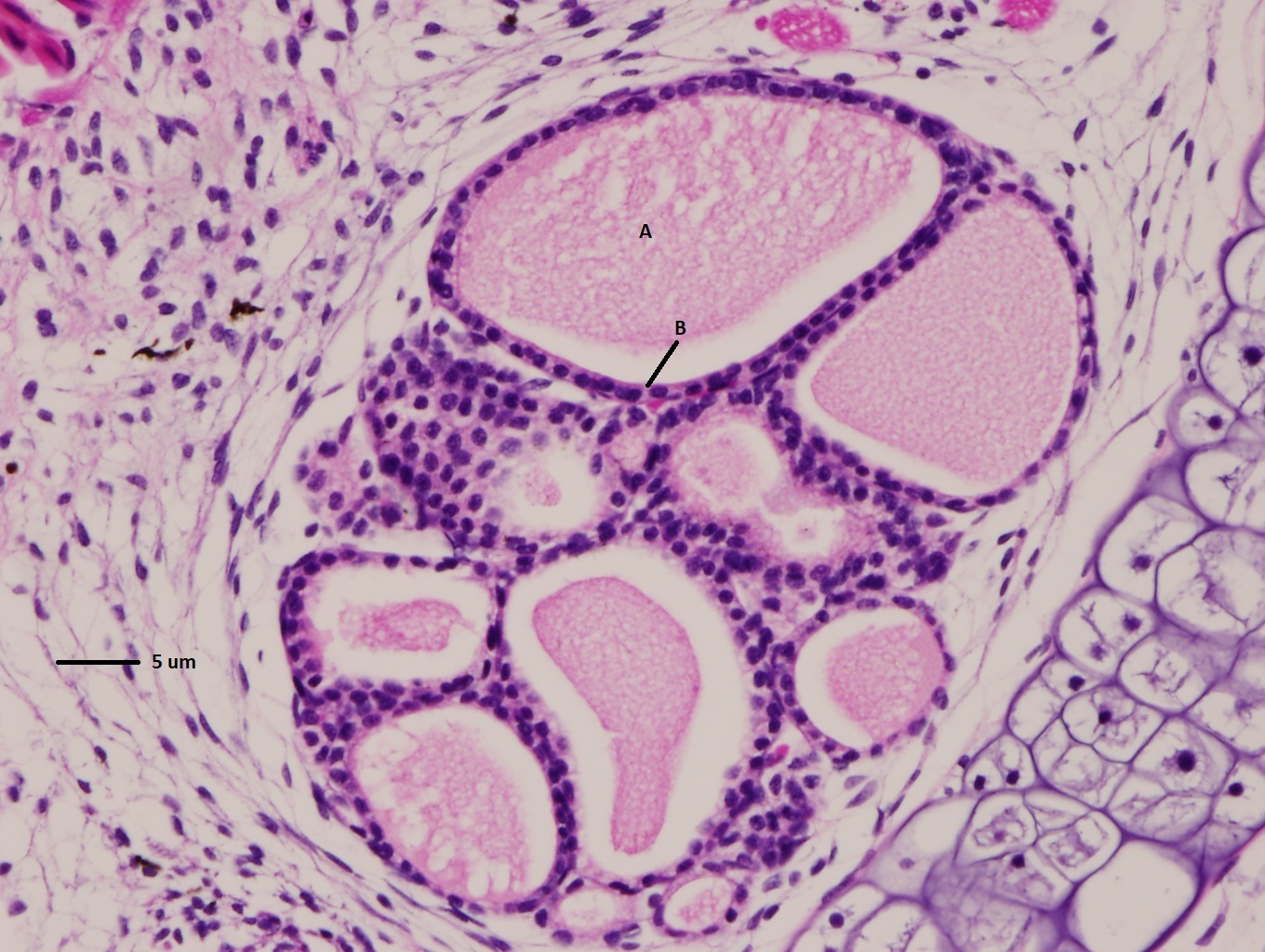


**Figure S2**. Toad thyroid photomicrograph. Gland is well developed, ovoid in transverse section. (A) Colloid present within follicle lumen; (B) Thyroid epithelium with well defined, condensed nuclei.


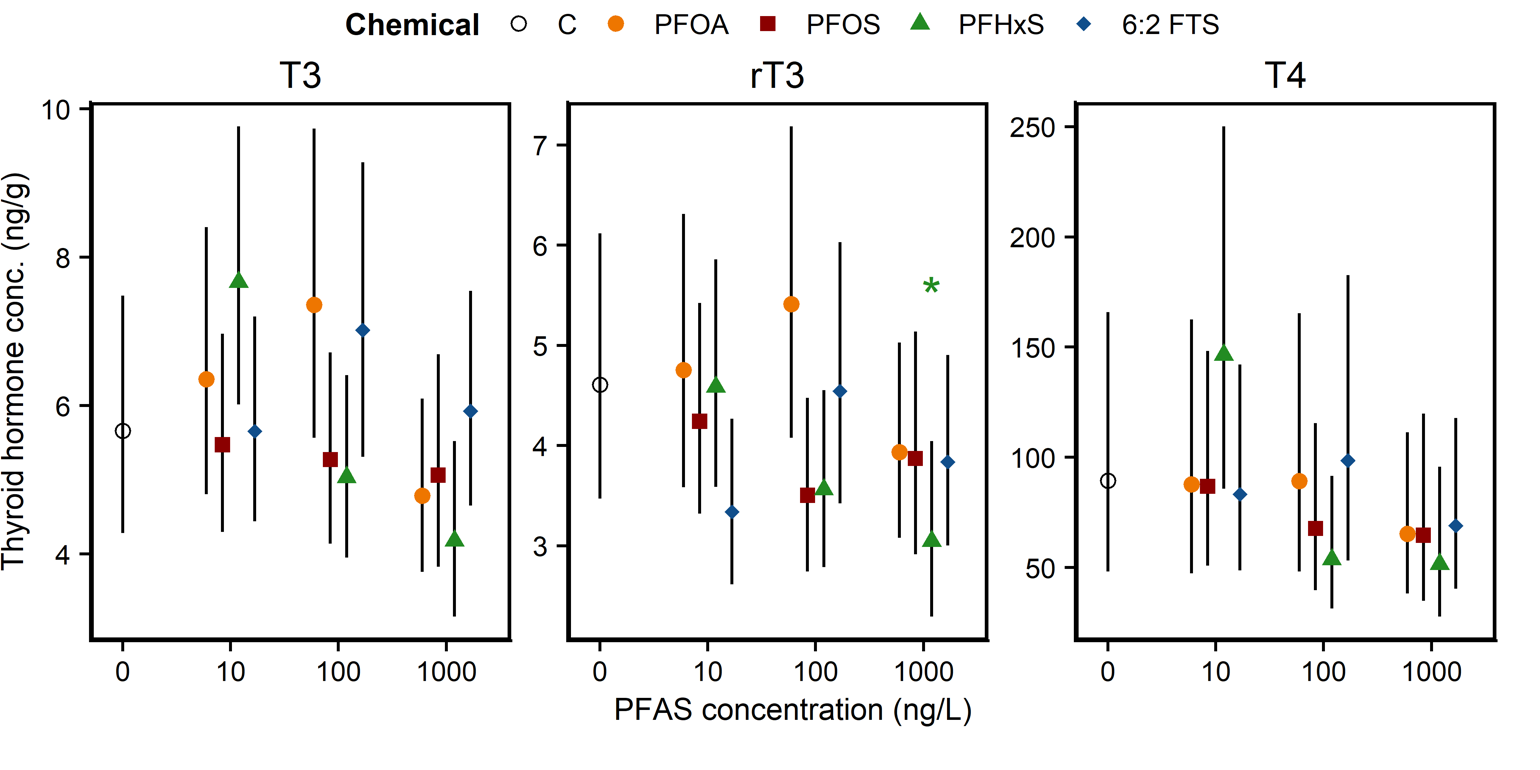


**Figure S3**. Mean thyroid hormone concentrations (95% CI) in whole body homogenates of toads at the climax of metamorphosis. Overall, there was limited evidence PFAS exposure affected thyroid hormone levels in toads, with the exception of the 1000 ng/L PFHxS treatment, which reduced mean rT3 levels by 27% relative to the control.
